# Brain viral reservoir seeding and neurological metabolic dysregulation in early‐life immunodeficiency virus infection

**DOI:** 10.1111/bpa.70104

**Published:** 2026-04-28

**Authors:** Li Ma, Robert Blair, Yao‐Zhong Liu, Xiaolei Wang, Eunice Vincent, Christopher Yu, Lara A. Doyle‐Meyers, Cissy Zhang, Ahmad Saied, Anne Le, Ronald S. Veazey, Qigui Yu, Huanbin Xu

**Affiliations:** ^1^ Tulane National Biomedical Research Center (TPBRC) Covington Louisiana USA; ^2^ Department of Pathology and Laboratory Medicine Tulane University School of Medicine New Orleans Louisiana USA; ^3^ Department of Biostatistics and Data Science Tulane Celia Scott Weatherhead School of Public Health and Tropical Medicine New Orleans Louisiana USA; ^4^ Department of Microbiology and Immunology Indiana University School of Medicine Indianapolis Indiana USA; ^5^ Gigantest Baltimore Maryland USA

**Keywords:** brain pathology, early treatment, HIV/SIV, infants, metabolic alterations, viral reservoirs

## Abstract

While viral reservoirs establish rapidly in the peripheral and lymphoid tissues in HIV‐exposed and infected infants, the timing and consequences of viral seeding in the developing brain remain unclear. Using a neonatal rhesus macaque SIV model, we tracked early brain‐associated viral DNA/RNA, neuropathology, and cerebrospinal fluid (CSF) metabolism, and evaluated the impact of early antiretroviral therapy (ART). CSF viral RNA was detectable by 2 days post infection (dpi), mirroring plasma viral load kinetics. In contrast, brain tissue SIV DNA and RNA remained largely undetectable through the first 3 days of infection. Despite this early viral dissemination, we observed no significant neuropathological lesions or CSF proinflammatory cytokine responses during the first week. However, marked CSF metabolic dysregulation emerged by 1 month post infection. Initiating ART early, specifically at 3 dpi, normalized these neurometabolic abnormalities. These findings demonstrate that CSF metabolomic shifts can occur independently of overt neuroinflammation during the early stages of viral seeding. These results support initiating ART as early as possible after perinatal HIV exposure to prevent the brain viral reservoir seeding and limit long‐term neurological sequelae.

## Graphical abstract

Brain viral reservoirs were undetectable in neonatal SIV during the first 3 days post infection; antiretroviral therapy (ART) at 3 dpi prevented brain reservoir seeding and normalized metabolic function, highlighting the necessity of very early treatment to protect the developing CNS.

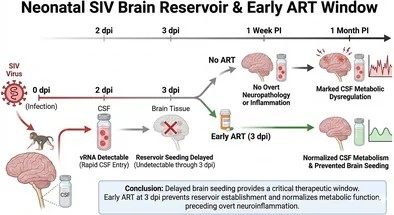

## 1 INTRODUCTION

Despite the recommendations for maternal antiretroviral therapy (ART), cesarean delivery, and formula feeding, which have significantly reduced vertical transmission rates from 25% to <2%, pediatric HIV acquisition remains a global challenge. Approximately 150,000 children are newly infected each year (UNAIDS, 2022) (1). In perinatally infected infants, viral reservoirs are established rapidly, leading to accelerated disease progression and a high risk of viral rebound upon treatment interruption (2, 3, 4, 5, 6, 7, 8, 9, 10, 11, 12). Our recent work demonstrated that in neonatal macaques infected within 24 h of birth, both plasma viral load and SIV RNA/DNA in systemic and lymphoid tissues are detectable as early as 1 day post inoculation (dpi). Notably, a 9‐month daily combined ART regimen (FTC/TFV/DTG, cART), initiated at 3 dpi, resulted in ART‐free viral remission in 80% of infant macaques (13). These findings suggest that sustained virological remission may be achievable through very early ART intervention in the pediatric population.

HIV and SIV can enter the central nervous system (CNS) through the “Trojan horse” mechanism, wherein infected monocytes traffic across the blood–brain barrier (BBB) and subsequently differentiate into long‐lived perivascular macrophages and microglia (14, 15, 16). As a distinct anatomical and immunological compartment, the CNS functions as a viral sanctuary site. Of these, early CSF viral load detected likely represents BBB leakage rather than brain reservoir establishment. Viral persistence is maintained even under suppressive ART, due to the limited CNS penetration of ARV drugs, restricted local immune surveillance, and the inherent longevity of infected myeloid lineage cells (17, 18, 19). In the adult infection model, viral reservoirs have been detected in the brain as early as 1–2dpi, demonstrating that CNS invasion can occur extremely rapidly following systemic exposure (20, 21, 22, 23). However, the dynamics of early CNS viral reservoir establishment in neonates remain poorly defined. The developing brain differs fundamentally from the adult CNS in terms of blood–brain barrier maturation, immune cell composition, microglial activation states, and metabolic demands (24, 25). Neonatal macaques closely recapitulate key aspects of human infant brain development and immune ontogeny, serving as a highly relevant model for studying perinatal HIV infection (26, 27). Because early viral dissemination in the systemic and lymphoid tissues of neonates diverges from patterns observed in adults, it remains unclear whether viral seeding of the developing brain follows similar temporal and mechanistic trajectories. Defining the earliest events of CNS viral entry and reservoir seeding is therefore critical for optimizing the timing of pediatric ART. Such precision is essential to prevent reservoir formation and encephalitis, thereby supporting healthy neurodevelopment in children. Beyond serving as viral reservoirs, the CNS is highly vulnerable to metabolic perturbations during early development. Emerging evidence from adult HIV infection indicates that neurometabolic abnormalities, affecting energy metabolism, mitochondrial function, lipid homeostasis, and neurotransmitter pathways, can be detected in cerebrospinal fluid (CSF) even in the absence of overt neuroinflammation or structural neuropathology (28, 29). These findings suggest that metabolic dysregulation may serve as an early, sensitive indicator of CNS involvement. However, it remains unknown whether such alterations occur during the hyper‐acute stages of perinatal infection.

In this study, we characterized the dynamics of early brain‐associated viral reservoir seeding in neonatal macaques infected with SIV at birth, alongside associated neuropathological and inflammatory responses. We also analyzed CSF metabolites in 1‐month‐old infants as a cumulative downstream consequence, comparing cohorts where ART was initiated at 3, 4, or 5 dpi, as a mere two‐day delay in treatment has been shown to preclude viral remission after analytical treatment interruption (ATI) (30). Our results demonstrate that both viral DNA and RNA remain undetectable in brain tissues until after 3 dpi, a timeline distinct from the rapid seeding observed in peripheral tissues. Furthermore, we observed no significant neuropathological changes or proinflammatory CSF responses during the first week of infection. Notably, early ART initiation largely normalized CSF profiles. Collectively, these findings highlight the importance of timely ART initiation in mitigating the impact of HIV exposure and/or infection on CSF metabolic abnormalities in infants, as assessed at 28dpi.

## 2 MATERIALS AND METHODS

### 2.1 Ethics statement

All animals in this study were housed at the Tulane National Biomedical Research Center in accordance with the Association for Assessment and Accreditation of Laboratory Animal Care International standards. All studies were reviewed and approved by the Tulane University Institutional Animal Care and Use Committee under an approved protocol. Animal housing and studies were carried out in strict accordance with the recommendations in the Guide for the Care and Use of Laboratory Animals of the National Institutes of Health (NIH, AAALAC #000594). All clinical procedures were carried out under the direction of a laboratory animal veterinarian. All procedures were performed under anesthesia using ketamine, with or without dexdomitor, or tiletamine hydrochloride, and analgesics (buprenorphine) were administered for all potentially painful procedures. All efforts were made to minimize stress, improve housing conditions, and provide enrichment opportunities (e.g., objects to manipulate in cages, varied food supplements, foraging and task‐oriented feeding methods, interaction with caregivers and research staff ).

### 2.2 Animals and samples

A total of 36 Indian‐origin rhesus macaques (*Macaca mulatta*), comprising 19 males and 17 females, were included in this study. Newborn (*n* = 26) and juvenile (*n* = 6) macaques were intravenously infected with 100 TCID50 SIVmac251. 4 age‐matched, 1‐month‐old animals served as SIV‐naïve controls. To characterize early infection, SIV‐infected newborns were euthanized at 1 day post infection (dpi; *n* = 3), 2 dpi (*n* = 3), 3 dpi (*n* = 6), 5 dpi (*n* = 2), and 7 dpi (*n* = 3). 9 neonatal animals received a 1‐month course of early combined antiretroviral therapy (cART; FTC/TFV/DTG) initiated at 3 dpi (*n* = 3), 4 dpi (*n* = 3), or 5 dpi (*n* = 3). Juvenile animals were euthanized for brain tissue collection during the chronic stage of infection (>3 months post SIV infection). Group sizes were determined based on a prioritized sampling strategy: larger cohorts were allocated to critical inflection points of viral reservoir seeding to account for biological variance, while minimizing animal numbers at the other time points in accordance with ethical guidelines for nonhuman primate research. Plasma, cerebrospinal fluid (CSF), and brain tissues previously collected (13, 31) were retrospectively analyzed to characterize brain viral reservoir seeding, neuropathology, and metabolic alterations.

### 2.3 Nucleic acid extraction of plasma, CSF and brain tissues

Brain tissues (Frontal cortex, Hippocampus and Basal ganglia) were processed for extraction of genomic DNA and total RNA using the AllPrep DNA/RNA Mini Kit (Cat No: 80311, Qiagen). In brief, ~50 mg of tissue was homogenized in 700 μL Buffer RLT Plus supplemented with 80 μL Proteinase K solution (20 mg/mL, Invitrogen, Cat. No. 2935092), followed by DNA and RNA isolation according to the manufacturer's instructions. Viral RNA from plasma and CSF was extracted using the QIAamp Viral RNA Mini Kit (Qiagen, Cat. No. 52962). All DNA and RNA samples were stored at −80°C until use.

### 2.4 Quantification of viral load and brain tissue‐associated SIV DNA/RNA

Specific primer and probe sets targeting the SIV *gag* region (13, 30), spliced SIV *tat*, and rhesus RPPR30 were synthesized by integrated DNA technologies. The sequences were as follows: SIV gag: Forward GTC TGC GTC ATC TGG TGC ATT C; Reverse CAC TAG GTG TCT CTG CAC TAT CTG TTT TG; and probe FAM‐CTT CCT CAG TGT GTT TCA CTT TCT CTT CTG CG‐BHQ‐1. SIV env: Forward CCT CAA TAA AGC CTT GTG TAA AAT TAT C; Reverse GTT GTT GAT GAT TTT GTC AAT CCC; and probe VIC‐TGC ATT ACT ATG AGA TGC‐MGB‐NFQ. Spliced SIV tat: Forward GAA CTC CGA AAA AGG CTA AGG CTA ATA CA; Reverse CCG TCT CCT TCT TTG CCT TCT CTG GTT; and probe ABY‐CTG CAT CAA ACA ACA GAC CCA TAT CCA ACA GGA CC‐QSY. Rhesus RPPR30: Forward TCA GCA TGG CGG TGT TT; Reverse GCT GTC TCC ACA AGT C; and probe VIC‐TTC TGA CCT GAA GGC TCT GCG C‐3IABkFQ‐1. The SIV gag primer/probe set was used to determine viral load in plasma and CSF (LOD, limit of detection, 30 copies/mL). Viral RNA from plasma and CSF was reverse transcribed into cDNA using the ThermoFisher SuperScript™ VILO™ Master Mix (Invitrogen, Grand Island, NY). Reactions were performed on a thermocycler at 25 °C for 10 min, a 42 °C for 60 min, and enzyme inactivation at 85 °C for 5 min, according to the manufacturer's instructions. Both cellular DNA (SIV gag and env) and cDNA (SIV gag and spliced tat) were quantified by digital PCR on the QuantStudio Absolute Q Digital PCR System (Applied Biosystems, Thermo Fisher, US) using the Absolute Q™ MAP16 Plate Kit and Master Mix. Cycling conditions were: 96 °C for 10 min, followed by 40 cycles of 96 °C for 15 s and 60 °C for 30 s. For brain tissue samples, cellular input was normalized by quantifying RPPR30 genomic copies (two copies per rhesus macaque cell). Quantification of SIV DNA/RNA were performed using a minimal of 2 × 10^5^ cell number equivalents per sample, and results were expressed as copies per one million cells (LOD, 5 copies per million cells). For samples with undetectable or very low SIV copy numbers, nested quantitative digital PCR, capable of detecting single‐copy target, was further performed to confirm true absence or accurately quantify low‐level copies.

### 2.5 Cytokine assay

Proinflammatory cytokines in plasma and CSF were measured by Luminex 200 systems (Bio‐Rad Inc., Hercules, CA, USA) according to the manufacturer's instructions, as we previously described (31, 32). Cytokine levels were measured using the ProcartaPlex NHP cytokine/GF37plex (Invitrogen). Briefly, 50 μL diluted plasma (1:1) was incubated with antibody‐coupled beads. Complexes were washed, incubated with biotinylated detection antibody, and then streptavidin‐PE. Cytokine levels were determined using a multiplex array reader from Luminex™ Instrumentation System (Bio‐Plex Workstation from Bio‐Rad Laboratories). Analyte concentrations were calculated using software provided by the manufacturer (Bio‐Plex Manager Software).

### 2.6 Brain tissue pathological assessment

Brain tissue sections were collected from neonatal macaques euthanized at 1–7 dpi following SIV infection. Gross pathological observations included the presence, location, size, number, and distribution of lesions. Samples were fixed in buffered 10% formalin with ionized zinc (Z‐Fix™, Anatech LTD, Battle Creek, MI), embedded in paraffin, and sectioned at 5 μm for routine hematoxylin and eosin (H&E) staining. Pathological evaluation was performed by a board‐certified veterinary pathologist (ACVP‐certified) blinded to infection status and cohort on multiple brain regions (frontal cortex, temporal cortex, parietal cortex, occipital cortex, basal ganglia, thalamus, hippocampus, cerebellum, and brainstem) and included assessment for each of the following lesions: cortical and perivascular mineralization, hemorrhage, ependymal loss, and microhemorrhage (no other lesions were noted across the cohort). Each lesion was scored in each section independently as follows: 0 (absent), 1 (minimal), 2 (mild), 3 (moderate), and 4 (severe). Cumulative pathology scores were generated for each animal by summating the lesions scores across all sections for an animal resulting in the following scoring scale: 0 (absent), 1–5 (minimal), 6–10 (mild), 11–15 (moderate), and >15 (severe), reflecting the overall severity of brain pathology. Based on the number of lesions scored and sections examined, the scoring system has a range from 0 to 180. Digital slides were viewed and imaged using HALO v3.6 (Indica Labs).

### 2.7 Metabolomics analysis of cerebrospinal fluid samples

Cerebrospinal fluid (CSF) was collected by cisterna magna puncture under aseptic conditions, immediately placed on ice, and centrifuged at 2000×*g* for 10 min at 4°C to remove cells. Supernatant aliquots were stored at −80°C until analysis. As previously described (33, 34), for metabolite extraction, 100 μL of cell‐free CSF was transferred to a 1.5‐mL centrifuge tube and mixed with 400 μL cold extraction solvent (acetonitrile: methanol,1:1 v/v) containing an internal standard mixture (stable‐isotope labeled standards; final concentration 0.02 mg/mL for each standard) was added. Samples were vortexed for 30 s and sonicated in an ice‐cooled bath (≈5°C, 40 kHz) for 30 min. Proteins were precipitated by incubation at −20°C for 30 min, followed by centrifugation at 13000×*g* for 15 min at 4°C. The supernatant was collected and evaporated to dryness under a gentle stream of nitrogen. Dried extracts were reconstituted in 100 μL of acetonitrile: water (1:1, v/v), sonicated for 5 min at low temperature, and centrifuged at 13,000×*g* for 10 min at 4°C. Final supernatants were transferred to LC–MS/MS vials (optionally passed through a 0.2 μm centrifugal filter) for analysis. A pooled quality control (QC) sample was prepared by combining equal aliquots of all study samples and processed alongside study samples. Chromatographic separation was performed on column (Waters Acquity BEH C18, 2.1 × 100 mm, 1.7 μm) at 40°C with a flow rate of 300 μL/min. Mobile phases were (A) water +0.1% formic acid (v/v) and (B) acetonitrile +0.1% formic acid (v/v). Injection volume was 3 μL and the total run time was 10 min. Mass spectrometric detection was performed on a Q Exactive™ Plus Hybrid Quadrupole‐Orbitrap™ Mass Spectrometer with Vanquish Duo UHPLC systems (ThermoFisher Scientific). Raw data were pre‐processed using Progenesis QI software (Waters Corporation, Milford, USA) for alignment, peak picking, and initial identifications and then further analyzed in R (Version 4.2.2) with Bioconductor packages. Metabolite identities were confirmed where possible against authentic standards or spectral libraries. Statistical testing used Benjamini–Hochberg FDR correction (FDR <0.05 considered significant). Visualization of significant metabolites was done via heatmaps using the pheatmap R package. Intensities were z‐score normalized, and hierarchical clustering was applied (both samples and metabolites). To explore relationships between metabolite classes, individual metabolites, and metabolic pathways (for upregulated vs. downregulated metabolites), Sankey diagrams were created using the networkD3 package.

### 2.8 Statistical analysis

Statistical analyses were performed by the non‐parametric Mann–Whitney *U* test using GraphPad Prism 10.3 Software (GraphPad Software, San Diego, CA). A nominal α level of 0.05 was used to define statistical significance, and the data are presented as mean and SEM.

## 3 RESULTS

### 3.1 Delayed establishment of brain viral reservoirs in neonatal macaques following SIV infection at birth

Neonatal brain tissues, including basal ganglia (BG), frontal cortex (FC), and hippocampus (HC), were collected post SIV infection (Figure 1A). In SIV exposed and infected newborn macaques, rapid viral replication was evident, with plasma viral load detectable in two of four animals at 1 dpi and in all neonates at 2 dpi. In contrast, viral load in cerebrospinal fluid (CSF) displayed a delayed pattern, remaining undetectable in some animals at 3 dpi (Figure 1B, C). In contrast to readily detectable viral genes in peripheral and lymphoid tissues, both cell‐associated SIV RNA and SIV gag or env DNA were undetectable in these neonatal brain tissues within the first 3 days post SIV infection (Figure 1E,F). These patterns were essentially consistent with the detection of virus replication‐associated spliced SIV *tat* RNA in these brain tissues (Figure 1D), as spliced SIV *tat* RNA reflects active viral replication rather than contamination from virus inoculum or circulating virions (35, 36). Collectively, these findings indicate that while rapid viral replication and dissemination occur in systemic and lymphoid tissue compartments of neonates (13), viral reservoirs are not consistently detected in neonatal brain prior to 3 dpi. This temporal delay, distinct from reservoir seeding in systemic and lymphoid tissues, provides a critical window in which timely pediatric ART could prevent the brain viral reservoir seeding, thereby limiting long‐term neurological consequences in growing children.

**FIGURE 1:**
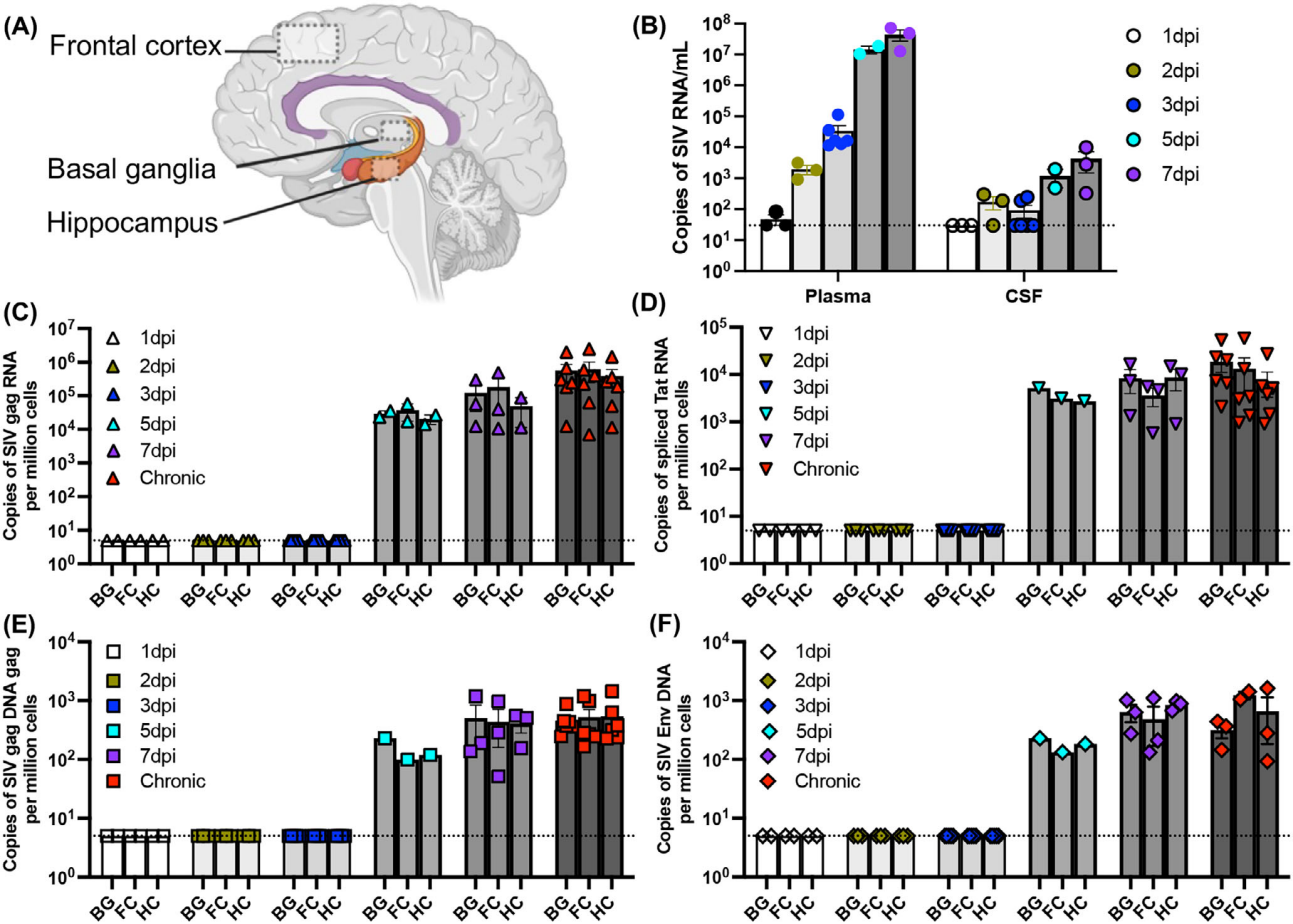
Early brain viral reservoir seeding in neonatal macaques infected with SIV within 24 h of birth. (A) Schematic representation of brain tissue collection sites for brain viral reservoir detection in SIV‐infected neonatal macaques. (B) Viral loads in plasma and cerebrospinal fluid (CSF) samples collected during the first week post infection. Sample size at each time point: 1 dpi, *n* = 3; 2 dpi, *n* = 3; 3 dpi, *n* = 6; 5 dpi, *n* = 2; 7 dpi, *n* = 3. (C–F) Levels of total SIV *gag* RNA (C), spliced SIV *tat* RNA (D), and SIV DNA targeting SIV gag or Env region (E and F) in the basal ganglia (BG), frontal cortex (FC), and hippocampus (HC) of neonatal macaques during the first week post infection, compared with brain tissues from chronically SIV‐infected juveniles (chronic, *n* = 6). Sample size at each time point for brain tissue analyses: 1 dpi, *n* = 2; 2 dpi, *n* = 3; 3 dpi, *n* = 6; 5 dpi, *n* = 2; 7 dpi, *n* = 3; chronic, *n* = 6. Note that spliced SIV *tat* RNA represents actively transcribed viral RNA, rather than residual inoculum. CSF viral load was detectable as early as 2dpi, whereas brain viral reservoirs were not consistently detected until after 3 dpi. Error bars represent the mean ± SEM.

### 3.2 Limited proinflammatory responses in neonatal macaques infected with SIV at birth

We previously reported that plasma cytokine levels do not significantly increase during acute SIV infection in infants compared with adults (31, 32). To further characterize proinflammatory responses in the neonatal CSF, particularly during the first week following SIV infection, we comparatively measured proinflammatory cytokine and chemokine levels in both plasma and CSF samples. As shown in Figure 2A,B, these typical proinflammatory mediators, including IL‐8, TNF‐α, G‐CSF, and MIP‐1β, did not differ significantly across time points or between plasma and CSF post infection, consistent with findings for other cytokines and chemokines included in the kit. Taken together, these results indicate that very early proinflammatory responses in both plasma and CSF are limited in neonatal macaques infected with SIV at birth, which may mitigate innate immune activation, meningitis, and potential brain pathology.

**FIGURE 2:**
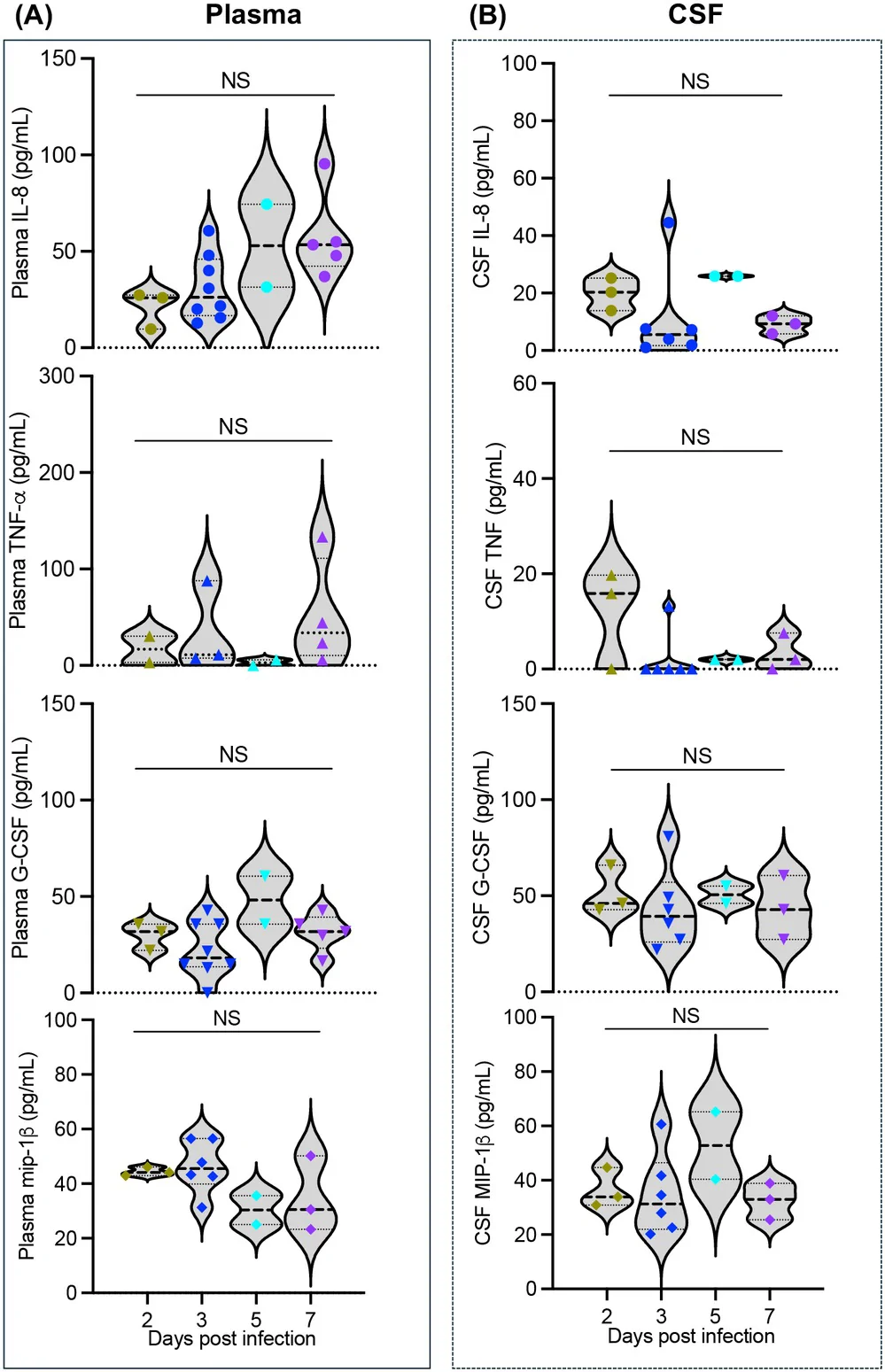
Proinflammatory mediator dynamics in plasma and CSF of neonatal macaques during the first week post SIV infection. (A, B) Changes of representative proinflammatory cytokines and chemokines—IL‐8, TNF‐α, G‐CSF, and MIP‐1β—measured in plasma (A) and CSF (B) during the first week after SIV infection. Sample sizes for each cytokine at each time point are provided in Table S1. Sample availability varied by analyte and time point due to limited plasma and CSF sample volume from neonatal animals. NS indicates no significant difference (*p* > 0.05). Error bars represent mean ± SEM.

### 3.3 Minimal brain pathological changes in neonatal macaques during early SIV infection

In parallel with assessing the dynamics of brain viral reservoir establishment in neonatal macaques during the first week following SIV infection, we comprehensively evaluated neuropathologic changes. Brain tissue sections, including but not limited to the frontal cortex, basal ganglia, and temporal cortex, were examined and independently scored for each of the following lesions: hemorrhage, infarction, cortical and perivascular mineralization, ependymal loss, inflammation, and degeneration. As shown by H&E staining (Figure 3A), no significant inflammatory lesions were detected in the brain sections within 7dpi, nor were there pathologies directly attributable to SIV infection. Minimal and scattered findings, such as focal cortical or perivascular mineralization, segmental ependymal cell loss, and microhemorrhages, were observed, but there was no evidence of degeneration, apoptosis, or necrosis. A standardized cumulative pathology score for each neonate, across all examined sections (0, absent; 1–5, minimal; 6–10, mild; 11–15, moderate; >16, severe), revealed minimal changes in 14 of 16 neonates and mild changes in 2 of 16 neonates with no pathology (inflammation, degeneration, apoptosis, or necrosis) during the first week of infection (Figure 3B). These findings indicate that brain pathology in neonates is scarce during very early SIV infection, consistent with the limited proinflammatory responses in CSF.

**FIGURE 3:**
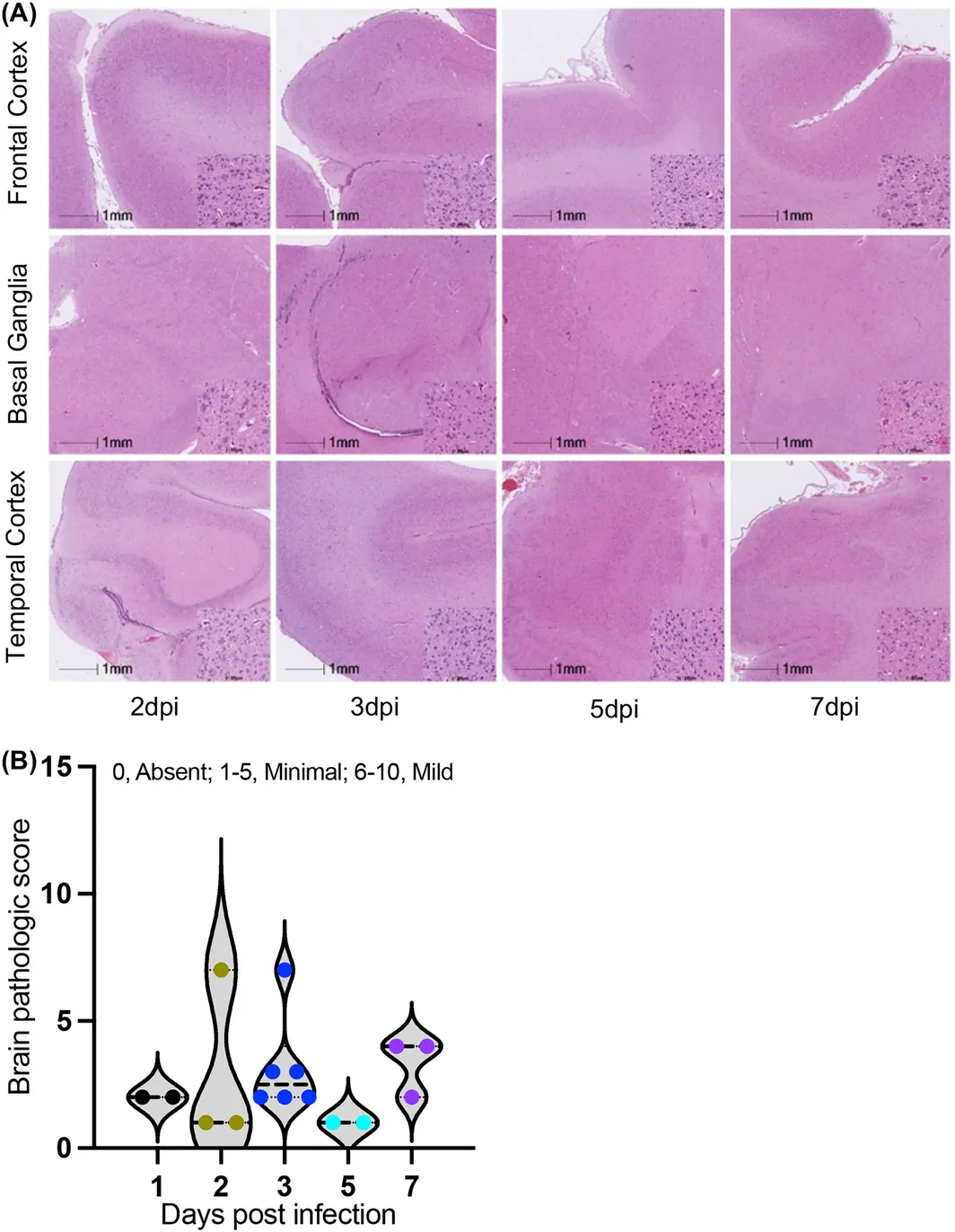
Histopathological assessment of brain tissues in neonatal macaques during the first week of SIV infection. (A) Representative H&E‐stained sections of the frontal cortex, basal ganglia, and temporal cortex from SIV‐infected neonatal animals collected at 2, 3, 5, and 7dpi. Scale bar = 1 mm. Note that significant pathology is not observed in brain regions examined during the first week of SIV infection. (B) Aggregate histopathologic scores across multiple brain regions (frontal cortex, temporal cortex, occipital cortex, basal ganglia, thalamus, hippocampus, cerebellum, and brainstem) during the first week post SIV infection. Sample size at each time point: 1 dpi, *n* = 3; 2 dpi, *n* = 3; 3 dpi, *n* = 6; 5 dpi, *n* = 2; 7 dpi, *n* = 3. Cumulative score system: 0, absent; 1–5, minimal; 6–10, mild; 11–15, moderate; >16, severe.

### 3.4 Reversal of CSF metabolic dysregulation induced by neonatal SIV infection through timely early antiretroviral therapy

Building on our previous findings that pediatric cART initiated at 3 days post‐infection (dpi) achieves sustained virologic remission, whereas a two‐day delay to 5 dpi results in viral rebound (13, 30). We assessed the impact of neonatal SIV infection and early ART on brain‐associated metabolism, we analyzed CSF samples collected from 1‐month‐old infant cohorts, grouped as SIV‐naïve (healthy), untreated SIV‐infected animals, and SIV‐infected with cART initiated at 3, 4, or 5 dpi. Partial least squares–discriminant analysis (PLS‐DA) revealed a clear separation between SIV‐naïve and untreated groups along the first major component (~30% of variance), while the 3‐dpi cART group closely mirrored the SIV‐naïve baseline (Figure 4A). Variance along the second component likely reflects individual differences and ARV‐specific effects. We note that cross‐validation yielded negative *Q* ^2^ values (−0.43 and −1.78) and unsupervised principal component analysis (PCA) showed substantial group overlap (Figure S1), indicating that while PLS‐DA models lack robust predictive power, they effectively highlight subtle, treatment‐driven metabolic shifts. At the individual metabolite level, untreated SIV infection induced profound systemic disruptions. Heatmap analysis displayed an enrichment of markers associated with severe energy dysregulation, oxidative stress, and neuroimmune suppression (e.g., phosphocreatine, sorbitol/dulcitol, kynurenine, norepinephrine, ornithine, and dihydrouracil). Conversely, essential neuroprotective factors and energy substrates, including bile acids, glucose, mannose, acetate, and key nucleotides, were notably depleted (Figure 4B). Sankey mechanically linked these alterations to metabolic encephalopathy, illustrating a severe catabolic state driven by hyperactivated tryptophan and galactose metabolism paired with a collapse in bioenergetics and nucleotide synthesis. Crucially, early cART reversed these pathological trajectories in a time‐dependent manner (Figure 4C, D). Treatment initiated at 3 dpi most effectively normalized both enriched and depleted metabolites toward healthy control levels, whereas initiation at 5 dpi yielded only partial restoration (Figure 4E). Collectively, timely early cART substantially reverses SIV‐induced CSF metabolic disturbances, replenishing the energy substrates and neuroprotective compounds essential for CNS homeostasis.

**FIGURE 4:**
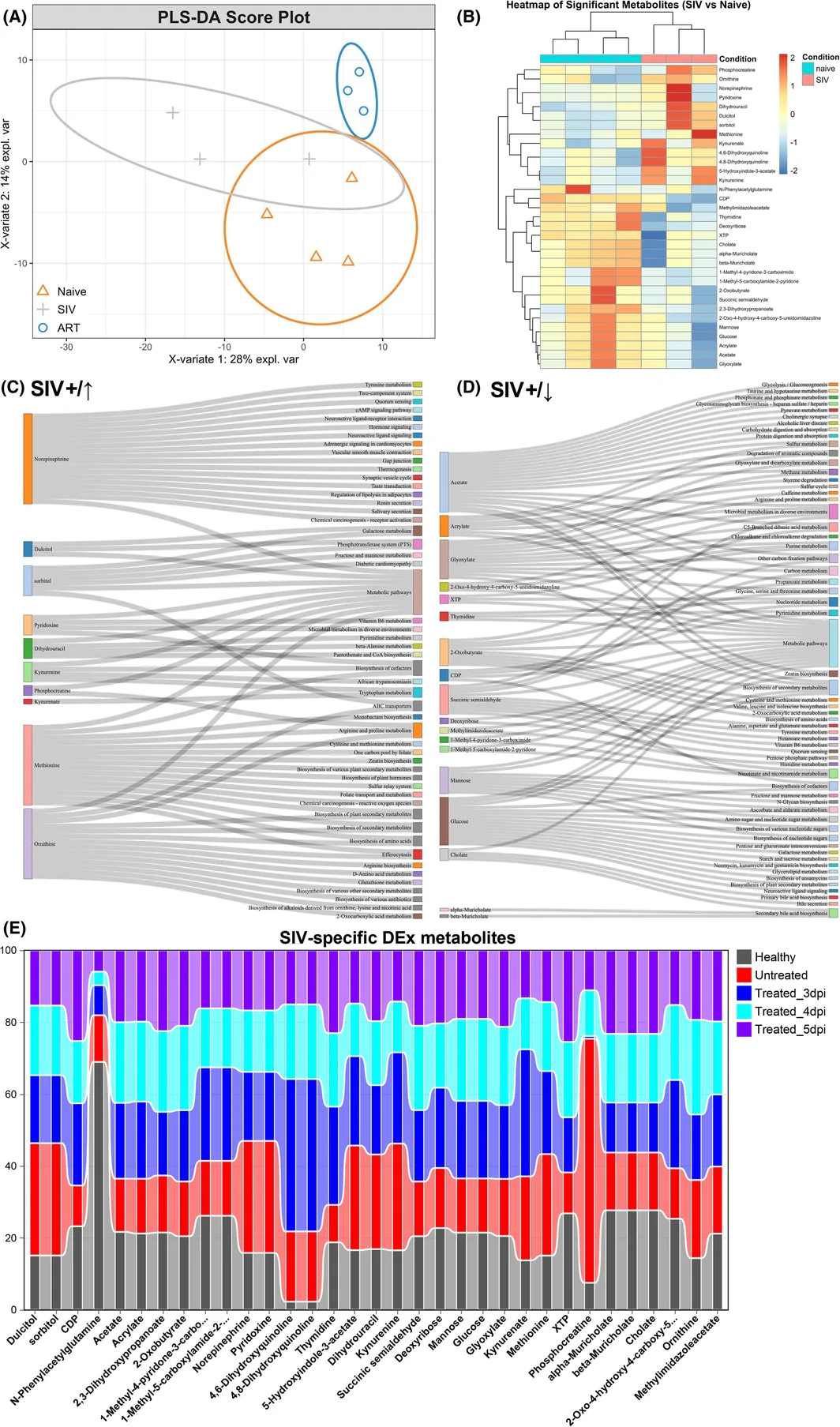
CSF metabolic alterations in neonatal macaques infected with SIV within 24 h of birth, with or without early treatment initiation. (A) PLS‐DA score plot showing distinct metabolic clustering across experimental animal groups. (B) Heatmap of significantly altered SIV‐specific metabolites in CSF from untreated SIV‐infected neonates, compared with SIV naïve controls. Rows represent metabolites, columns represent individual samples. Z‐score indicate relative metabolite abundance (scale bar: Red, upregulated; blue, downregulated). (C‐D) Sankey diagrams depicting SIV‐specific upregulated (C) and downregulated (D) metabolites and their associated biological pathways/functions. (E) Effect of early treatments on CSF metabolites, compared with both SIV naïve and untreated SIV‐infected neonates. A total of 34 SIV‐associated differentially regulated metabolites were identified and present here, most of which were restored by early treatment, particularly when initiated at 3 dpi. Animal groups included SIV naïve controls (*n* = 4), untreated SIV infected neonates (*n* = 3), and SIV infected neonates treated with 1‐month of cART starting at 3 dpi (*n* = 3), 4dpi (*n* = 3), or 5 dpi (*n* = 3). Newborn macaques (*n* = 12) were intravenously infected with an identical SIV inoculum, with CSF samples collected at 1 month post SIV infection. The data are shown as p‐adjusted metabolite intensity normalized at percentage scale across 5 cohorts.

## 4 DISCUSSION

Children exposed to and infected with maternal HIV frequently experience neurodevelopmental delays, cognitive impairment, and mental health disorders (37, 38, 39, 40). Yet, the impact of neonatal HIV infection at delivery on early brain reservoir seeding, encephalopathy, and cerebrospinal fluid (CSF) metabolic alterations, particularly in untreated versus early‐treated infants, remains poorly understood, largely due to ethical and practical limitations in obtaining brain samples. Utilizing a neonatal macaque model of SIV infection, we investigated viral reservoir establishment and neuropathological changes during the first week of infection and evaluated CSF metabolic profiles at 1 month of age in the presence or absence of early cART. Our findings show that brain SIV DNA and SIV RNA were not consistently detected until after 3 dpi, that neuropathology and neuroinflammation were minimal during this window, and that SIV infection caused profound CSF metabolic dysregulation that was largely restored by early treatment. These findings highlight the importance of initiating cART at the earliest therapeutic opportunity to preserve brain development and mental health in children living with HIV.

Understanding reservoir establishment in the neonatal CNS following maternal HIV infection is critical for developing strategies to mitigate neurological complications and optimize early prevention interventions. In adults, viral reservoirs are found in microglia, macrophages, astrocytes, and T cells across multiple brain regions (41, 42, 43, 44, 45, 46, 47), where intact proviruses persist despite long‐term ART (26, 48, 49). These reservoirs are seeded when infected cells migrate across the blood–brain barrier (BBB) (15, 20, 50, 51). Our previous study demonstrated that viral DNA, but not integrated proviral DNA, could be detected in systemic and lymphoid tissues of neonates as early as 1dpi (13). Here we showed that while SIV RNA was detectable in CSF by 2dpi, consistent with earlier infant macaque study (52), both SIV DNA and RNA were absent from brain tissues until after 3 dpi, as confirmed by digital PCR and nested digital PCR. Spliced SIV tat transcripts in the brain paralleled SIV gag RNA levels, reflecting active replication from local viral DNA rather than residual inoculum. Notably, as total SIV DNA remained below the limit of detection in brain tissues during the first 3 days post infection, performing integration and intact proviral DNA assays was technically unfeasible for these specific samples. Intact Proviral DNA Assay (IPDA) is constrained by the limited availability and nature of the samples. Specifically, tissue‐derived specimens, particularly from brain, contain low absolute numbers of potentially infected cells, and are more prone to DNA shearing following homogenization. These factors significant technical challenges for IPDA, which typically requires on the order of ≥5 × 10^5^ intact cells and high‐quality, minimally fragmented DNA to ensure reliable and interpretable results. While in situ hybridization (e.g., RNA/DNAscope) provides valuable spatial localization on tissue slides, PCR‐based analysis of homogenized brain tissue sections remains the gold standard for the ultrasensitive quantification of low‐copy nucleic acids. Further studies are planned to map the specific cell types and anatomical distribution of SIV‐infected cells across multiple brain regions in neonatal and infant macaques. Collectively, these findings provide a mechanistic basis for why initiating cART at 3 dpi in neonates can effectively suppress viral replication and achieve sustained virologic remission (13). In other studies where neonatal macaques were either infected with SIV within 24 h, 1 or 3 days after birth, SIV DNA/RNA in brain tissues remains essentially undetectable but could be consistently detected by 7dpi, accompanied by histopathological lesions at 14dpi and progressive reductions in brain growth thereafter (53, 54, 55). In contrast, older infant macaques (3 weeks to 5 months) exhibit earlier brain reservoir seeding, with SIV DNA detectable as early as 1‐2dpi (21, 53, 56). Despite detectable systemic viral dissemination, neonatal SIV infection during the first week caused only limited neuropathological changes. We observed no significant inflammatory lesions or degeneration in brain tissue sections and only minimal scattered changes (e.g., mineralization, microhemorrhages, segmental ependymal loss). Consistently, both plasma and CSF cytokine analyses showed limited proinflammatory responses, echoing prior reports of limited cytokine induction in infant macaques (31). This relative lack of early neuroinflammation may delay the onset of neuropathology compared to older animals. Interestingly, hippocampal neuronal loss has been reported to be more pronounced in neonates infected orally compared to intravenously (57), suggesting the contribution of infection route to neuropathogenesis. Together, these discrepancies in the timing of viral seeding and neuropathology between neonates and older infants are likely attributable to differences in age, blood–brain barrier permeability, abundance of brain target cells, and inoculation route (26, 54, 55, 57, 58, 59).

Due to small sample sizes, this study has limited statistical power; therefore, both multivariate (PLS‐DA) and univariate (e.g., Mann–Whitney) analyses are exploratory. PLS‐DA plots visualize group structure but lack predictive value, as evidenced by negative Q^2^ scores. Furthermore, unsupervised Principal Component Analysis (PCA) showed substantial group overlap (Figure S1), suggesting that treatment‐driven metabolic differences are subtle relative to overall variability. Future larger cohorts are required to confirm these exploratory findings. Despite these limitations, metabolomic profiling revealed profound CSF metabolic disturbances at 1‐month post SIV infection, as CSF metabolites are tightly linked to neurological development, cognition, and brain physiology (60, 61, 62, 63). Untreated neonates exhibited a metabolic crisis marked by energy substrate depletion (e.g., glucose, mannose, acetate, succinic semialdehyde), increased oxidative stress, and dysregulated neurotransmitters. Elevated markers of neuroinflammation and toxicity (kynurenine, norepinephrine, 5‐HIAA, and sorbitol/dulcitol) paired with reduced bile acids reflects a vulnerable state that risks long‐term brain developmental impairment (64, 65, 66, 67). Encouragingly, early cART largely reversed these imbalances in a timing‐dependent manner. Initiating treatment at 3 dpi most effectively restored metabolic homeostasis, consistent with our when early treatment is initiated at 3dpi (13, 30), whereas 5 dpi initiation yielded only partial restoration. Altered SIV‐related metabolites like phosphocreatine, sorbitol, and kynurenine, emerged as candidate biomarkers for disease severity and therapeutic efficacy. Ultimately, timely intervention in SIV‐infected neonates prevents brain reservoir seeding and restores metabolic homeostasis. We have identified distinct CSF metabolic alterations that appear to be directly associated with antiretroviral drug exposure per se. A more comprehensive and dedicated analysis of antiretroviral‐related metabolomic signatures, including plasma and lymphocyte compartments, is currently underway. These findings parallel clinical data in HIV‐infected infants (68, 69) and highlight the urgent need for immediate therapeutic intervention in HIV‐exposed neonates to protect long‐term brain health.

## AUTHOR CONTRIBUTIONS

Li Ma and Huanbin Xu contributed to writing the original draft, Robert Blair, Yao‐Zhong Liu, Xiaolei Wang, Eunice Vincent, Lara A. Doyle‐Meyers, Ahmad Saied, Ronald S. Veazey, Qigui Yu contributed to methodology, data analysis, data interpretation, and manuscript revision, Xiaolei Wang and Huanbin Xu were responsible for study design, supervision, and project administration.

## FUNDING INFORMATION

This study was supported by National Institutes of Health grants R01MH133474, R56NS129438, R01HD099857, U42 OD024282, U42 OD010568, and the Office of Research Infrastructure Programs (ORIP) grant no. OD011104. This study is also supported by NIH grant U2C‐DK119886 and OT2‐OD030544 grants.

## CONFLICT OF INTEREST STATEMENT

The authors declare no competing interests.

## Supporting information


**FIGURE S1.** Principal component analysis (PCA) score Plot.

**TABLE S1.** Sample sizes for each inflammatory mediator measurements.

## Data Availability

The metabolomics datasets have been deposited in a publicly accessible repository, Metabolomics Workbench: An international repository for metabolomics data and metadata, metabolite standards, protocols, tutorials and training, and analysis tools (2016) (70). This study is available at the NIH Common Fund's National Metabolomics Data Repository (NMDR) website, the UCSD Metabolomics Workbench, https://www.metabolomicsworkbench.org, where it has been assigned Study ID ST004722. The data can be accessed directly via its Project DOI: https://doi.org/10.21228/M8NZ8G.
